# Minimally invasive procedure for optic disc pit maculopathy: vitrectomy with scleral plug and analysis on pattern of resolution

**DOI:** 10.1038/s41598-023-42839-y

**Published:** 2023-09-21

**Authors:** Anadi Khatri, Saurav Man Shrestha, Gunjan Prasai, Kamal Pandit, Priya Bajgai, Rupesh Agrawal, Vishali Gupta

**Affiliations:** 1 Birat Eye Hospital, Biratnagar, Nepal; 2 Birat Medical College and Teaching Hospital, Biratnagar, Nepal; 3 Byers Eye Institute, Stanford University, Palo Alto, CA USA; 4 Kathmandu Eye Centre, Kathmandu, Nepal; 5 Tilganga Institute of Ophthalmology, Tilganga, Kathmandu, Nepal; 6 B.P Koirala Lions Centre for Ophthalmic Studies, Kathmandu, Nepal; 7 Nepal Eye Hospital, Kathmandu, Nepal; 8 National Healthcare Group Eye Institute, Tan Tock Seng Hospital, Singapore, Singapore; 9 Singapore Eye Research Institute, Singapore, Singapore; 10 Moorfields Eye Hospital, NHS Foundation Trust, London, UK; 11 Advanced Eye Centre, PGIMER, Chandigarh, India

**Keywords:** Visual system, Retina, Eye diseases, Optic nerve diseases, Retinal diseases, Vision disorders, Pathogenesis, Outcomes research

## Abstract

Optic disc pit maculopathy (ODP-M) is a rare complication of optic disc pit which can cause irreversible visual impairment. The aim of this study is to evaluate the anatomical and functional outcomes and pattern of resolution of ODP-M following vitrectomy with posterior vitreous detachment (PVD) induction and scleral tissue plug for treatment of ODP-M without ILM peeling, laser or use of long term gas/tamponade or head positioning. This retrospective study included 7 patients with ODP-M, meeting the inclusion criteria. Patients were followed up for 6 months. Complete anatomical success was defined as “Total resolution of all the fluid in retinal compartments”. All of the patients had complete resolution of the optic pit maculopathy following surgery. The mean duration for complete resolution was 18.3 weeks. Pattern of resolution of ODP-M was found to be resolution of the subretinal fluid (SRF) followed by disappearance of the retinoschitic lesions (RL) and finally disappearance of macular edema (ME). The proposed minimally invasive procedure (MIP) can produce comparably good and equally reliable results for the treatment of ODP-M.

## Introduction

Optic disc pit (ODP) is a rare congenital deformity of the optic nerve head—occurring approximately 1 in 11,000 births^1^. It is unilateral in more than 80% of the cases and has no predilection for gender^2^. Most of the cases of ODP are sporadic, however some literature advocate that unilateral cases may be inherited in an autosomal dominant pattern^3^. It has been regarded as a part of the spectrum of congenital optic disc cavitary anomalies which include optic disc coloboma, extra papillary cavitation, and morning glory anomaly^4^.

While isolated cases of ODP may be asymptomatic, it is estimated that up to two-thirds of the patients develop visual problems due to progressive accumulation of subretinal or intraretinal fluid causing serous macular detachment or schitic lesions—commonly known as ODP—maculopathy (ODP-M)^5,6^. There seems to be no predilection or specific triggering/predisposing factor for the development of ODP-M. However, many cases are reported to be associated with lamellar or full-thickness macular hole, macular cystic changes, or retinal pigment epithelium atrophy^7–9^.

Many surgical techniques have been proposed for ODP-M with varying success rates. These range from macular buckling, pars plana vitrectomy (PPV] with or without internal limiting membrane (ILM) peeling to a more recent introduction of the pit “plug” using internal limiting membrane, platelets, neurosensory retinal transplant and autologous scleral graft^10–16^. Various reports have stated the necessity of juxta papillary laser photocoagulation or internal tamponade but have been limited to surgeons’ own discretions with no hard and fast rule or clear-cut guidelines^11,17,18^.

With reference to the existing literature, where many of the above-mentioned techniques have been performed in various combinations, we performed only PPV with posterior vitreous detachment (PVD) induction and scleral tissue plug for treatment of ODP-M without ILM peeling, laser or use of long term tamponading agents in our surgeries. We here report the outcomes and propose a pattern of resolution in ODP-M with our minimally invasive procedure.

## Materials and methods

This is a retrospective study done at Birat Eye Hospital, Nepal. Records of the patients who underwent this minimally invasive procedure [MIP] for management of ODP-M from 2018 to 2020 were retrieved from the hospital data. All surgeries were performed by a single surgeon. Since the aim of the study is also to evaluate the pattern of the resolution, only the patients with ODP-M who presented with all three features—sub-retinal fluid (SRF), retinoschitic lesions (RL), and macular edema (ME) were included. A total of 7 patients meeting all the inclusion criteria were included in the study. Patients with lamellar macular holes, impending or established full-thickness macular holes, abnormalities associated with ILM surfaces like epiretinal membranes or macular puckering, or had other additive retinal pathologies and those who failed to complete at least 6 months of follow-up were excluded from the study. The study was approved by the medical advisory board of Birat Eye Hospital (BRT/2021/09/R101) and adheres to the declaration of Helsinki. Informed and written consent was obtained from all the subjects who participated in the study.

### Examination

All the patients included in the study had undergone a complete ocular examination which included best-corrected visual acuity (BCVA), intraocular pressure (IOP) examination, slit-lamp examination, and fundus evaluation after dilatation, and optical coherence tomography (OCT) at baseline and at follow-ups. Swept Source OCT (Swept Source DRI Triton Plus; Topcon, Japan) scans of 9 mm including imaging of both the fovea and optic disc were done. Parameters evaluated at each scan included SRF, RL and ME. The proprietary algorithm/software of Topcon (ImageNet and OCTARA) was used to produce and evaluate the results of the scan.

### Surgical technique

All the surgeries were performed under peribulbar anesthesia using 23-gauge vitrectomy systems (Associate 6000, Dutch Ophthalmic Research Centre (DORC), Netherlands.) After opening the 3 ports, the infusion was placed, and continuous irrigation was started with a bottle height of approximately 50 cm from the patient’s head level. Pars plana vitrectomy (PPV) was initiated from the core at 4500 cuts per min (CPM), with a peristaltic driven aspiration of 45 ml and vacuum of 200 mmHg. Vitreous staining was done using triamcinolone acetonide to identify the posterior hyaloid. Posterior hyaloid detachment and removal were done using venturi at 250 mmHg vacuum. Peripheral vitrectomy and shaving were done using 6000 CPM with a peristaltic driven aspiration of 25 ml/min and vacuum of 150 mmHg.

A 3 mm by 3 mm peritomy was done at approximately at 11 O’clock position, 2–3 mm from the limbus, and the underlying sclera was cauterized using the rapid movement of the cautery tip with light diathermy (30% power). A 1.6 by 2.2 mm of homologous partial-thickness scleral tissue was dissected and further trimmed to approximately 1 mm by 2 mm size. The tissue was grabbed at one end by retinal forceps (Eckardt End gripping 23G retinal forceps, DORC) and introduced into the vitreous cavity via the port usually designated for the cutter. The tissue was placed over the optic disc, which was then grasped just behind the leading edge and tucked into the pit. There is usually very little resistance while inserting the scleral tissue. Sometimes, the tissue may need to be trimmed for the appropriate size for which we advise it best to take the tissue out of the vitreous cavity and do it in an extra-ocular environment.

After inserting the leading edge of the tissue, the rest of the tissue can be grasped and pushed slowly. This can also be done by using the back or the side edge of the tip of one of the pincers while keeping the forceps open. The lagging end of the tissue can be tucked similarly. It is advised to keep check of the blood vessels or induced venous pulsations that might occur while introducing the scleral plug. In case of such an event, though very rare, we advise withdrawing the tissue slowly until the pulsations stop or the vessels refill. This usually means the plug might be larger and may require to be reduced in size.

With the scleral plug in situ, the surgery can be considered complete and the ports can be closed in a regular fashion. We did not perform ILM peeling, juxta papillary laser photocoagulation, or any other procedure in any of the cases. We performed fluid air exchange (FAX) in all of our cases and left them filled with atmospheric air (FAX pressure of 40 mmHg) at the end of the surgery.

The retina was evaluated on the first post-operative day and followed up 1 monthly up to 6 months postoperatively. A complete ocular evaluation was done on each follow-up as described above. The surgical video of the procedure is illustrated below (see Supplementary Video 1).

Criteria for success were adopted as per the study published by Babu et al.^12^ They defined complete anatomical success as “Total resolution of all the fluid in retinal compartments, that is, SRF and RL on OCT.”

## Results

The study included 7 eyes of 7 patients. Among those patients, 4 were male and 3 were female. The mean age was 27.43 ± 11.38 years (range 14–54 years). None of the patients had received any prior treatment before presenting to us.

All of the patients had complete resolution of the optic pit maculopathy following surgery. The mean duration for complete resolution was 18.3 weeks (± 4.6weeks). The mean central macular thickness (CMT) at the time of enrollment was 815 microns (± 112 microns). At the end of the follow-up, the mean CMT was found to be 298 microns (± 92 microns). The mean visual acuity of the operated eyes at the time of presentation and the end of the follow-up was found to be 0.58logMAR (± 0.12 logMAR) and 0.34 logMAR (± 0.12 logMAR) respectively.

**Figure 1:**
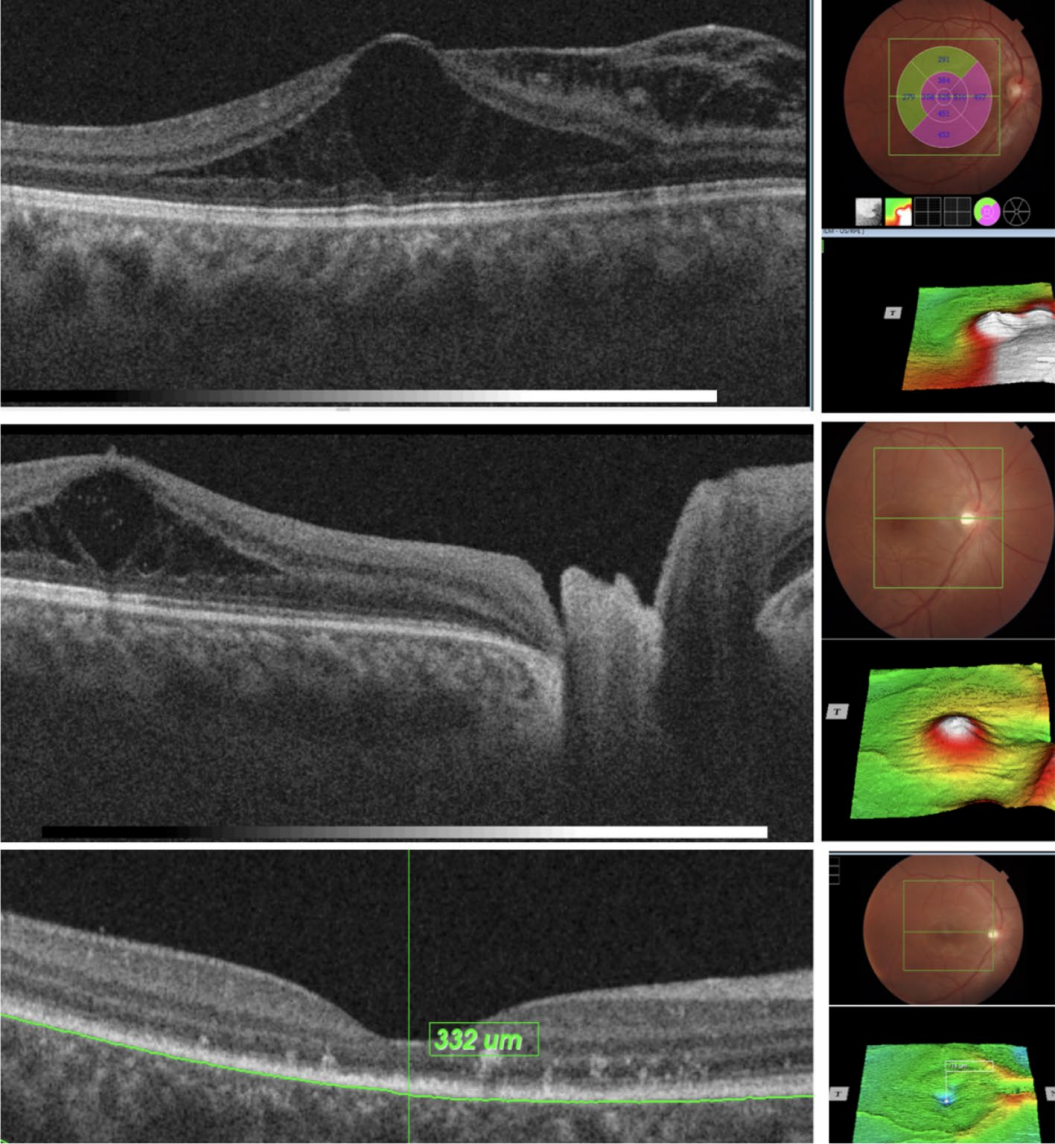
Pattern of resolution in a case of optic pit maculopathy following minimally invasive procedure. Subretinal fluid resolution and “retinoschitic” lesions were the first to resolve (top) followed by resolution of the residual cystoid macular edema (middle and bottom).

The overall resolution pattern is depicted in Fig. 1.

Resolution of the subretinal fluid was found to be the first clinical feature following the surgery. The resolution started from the temporal border of the optic nerve head. The mean duration at which the resolution completed was 4.7 weeks (± 2.1 weeks). It was found to start as early as 3.1 weeks and resolved as late as 7.9 weeks. The mean CMT was found to be 517 microns (± 67 microns). The mean visual acuity at the time of complete resolution of the subretinal fluid was 0.49 logMAR.

The resolution of the SRF followed by disappearance of the RL which was found to completely resolve at a mean duration of 9.2 weeks (± 5.8 weeks). The earliest resolution of RL occurred at 6.7 weeks but this process was found to occur up to 14.5 weeks after surgery. The mean CMT was found to be 431 microns (± 67 microns) at this stage where SRF was not observable in any of the operated eyes. The mean visual acuity was 0.43 logMAR.

**Figure 2:**
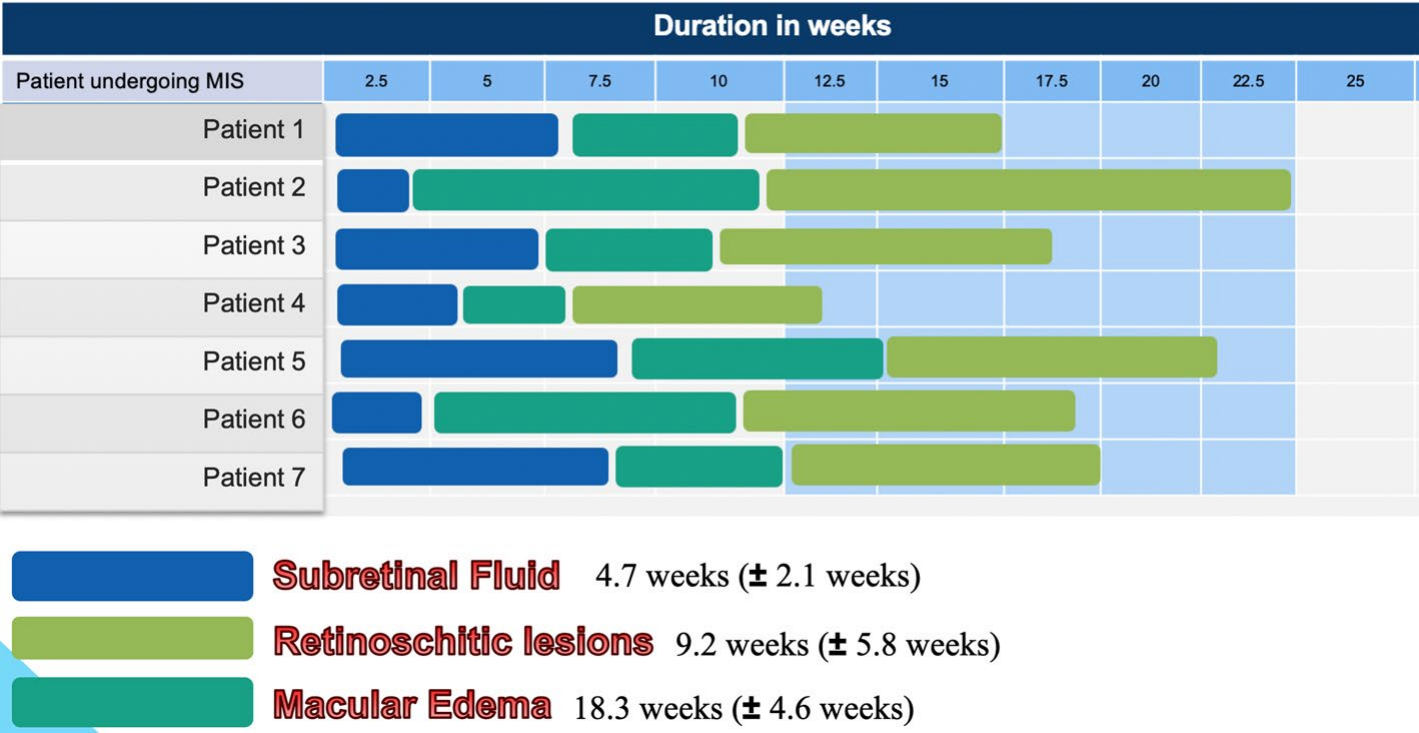
The chart shows the duration for resolution of Subretinal fluid, Retinoschitic lesions and macular edema in linear timeline for each patient. Blue bar representing resolution of subretinal fluid is the first in the sequence followed by green bar representing resolution of retinoschitic lesions. Macular edema was last to resolve in all patients taking up to 22.5 weeks post-surgery

The macular edema was found to be the last in the sequence to resolve. The mean duration at which it completely resolved was 18.3 weeks (± 4.6 weeks). The earliest duration at which the resolution of macular edema occurred was at 11.7 weeks, but it was found to extend up to 22.5 weeks post-surgery. The mean CMT was 298 microns (± 69 microns). The mean visual acuity at the time of complete resolution of the subretinal fluid was 0.34 logMAR (± 0.12 logMAR). The overall pattern of resolution and duration in all the cases are depicted in Fig. 2.

None of the eyes developed cataract or elevated intraocular pressure following surgery till the date of complete resolution of the maculopathy.

**Figure 3:**
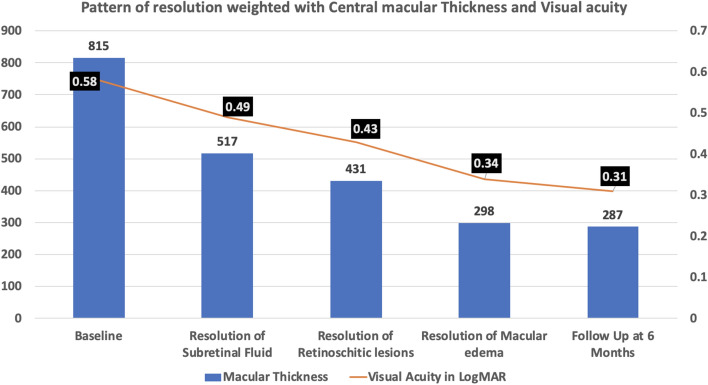
The chart shows the improvement in visual acuity and central retinal thickness from the baseline up to 6 months follow up. Mean visual acuity (logMAR) and mean central macular thickness (microns) shows further improvement with resolution of subretinal fluid, retinoschitic lesion and macular edema.

At 6 months, the mean visual acuity as 0.31 logMAR (± 0.08 logMAR), mean CMT was 287 microns (± 43 microns) (Fig. 3).

## Discussion

Visual loss due to ODP-M can be irreversible and disabling, due to persistent SRF and chronic macular detachment, if left untreated.

While various treatment modalities have been proposed and appraised by several researchers, the goal of the treatment remains the same—fabricating a barrier to prevent the migration of fluid that causes the serous macular detachment. Different surgical techniques have been shown to be effective in the literature, but we have been striving for an unsophisticated and accessible technique that is also equally reliable and reproducible in the simplest VR setup.

### Technique

We evaluated the outcomes of 7 eyes with ODP-M, who underwent MIP for ODP-M. This meant the patients only underwent pars plana vitrectomy with PVD induction with an autologous scleral flap plug. ILM peeling laser photocoagulation or long term tamponades were not performed in any of the cases. All the patients had successful anatomical and commendable physiological success within the follow-up period.

PVD induction has been enlisted as the most critical step disregarding to the adjunctive therapies. It has been postulated and also supported by various researchers that the creation of a PVD not only aids in relieving the traction but also facilitates the macular reattachment by decreasing both tangential and longitudinal forces^19–22^. We performed PVD induction in all cases along with scleral patch graft. Scleral graft, when tucked inside the optic pit, can perform like a capable barrier that prevents the seepage of fluid.

ILM peeling in patients with ODP-M is still disputed Many authors still consider it despite evidence of successful outcomes without the need for peeling of the ILM^14,17,23–25^. While some have suggested it to be an important step of the surgery, good results have been reported without performing ILM peeling- questioning its requirement for surgical success^11,13,21,22,26–28^. We did not perform ILM peeling in any of our cases and also achieved desirable and comparable results. It must however be remembered that we have not included complicated ODPM such as those with macular holes.

Gas tamponade has been performed in the vast majority of published cases, as it is used to create a temporary barrier blocking the passage of fluid through the ODP^29,30^. There have also been reports where gas tamponade was not used. VA had improved but the fluids did not resolve completely^11^. In our procedure we left the vitreous cavity with atmospheric air after the fluid air exchange. A successful attachment was achieved in all of the eyes which may be suggestive that only atmospheric air may be adequate without the requirement of long term expansile gases for good outcomes.

None of our eyes received endolaser. It has already been demonstrated that a laser may not be required^11,21^. Furthermore, we believe that lasering the retina on the temporal margin of the optic disc can damage the papillomacular bundle hence compromising the functional component of the retina even if the anatomical success is achieved.

Of all the possible combinations of the surgical step, our combination is very close to the one reported by Hirakata, Akito et al.^11^ where PPV with PVD induction was performed but the eyes did not receive laser, ILM peeling (except 1 in 8 cases due to surgical failure) or any special tamponade. However, the major difference is the use of scleral plug in our surgery. Although this does seem an additional step, the duration for the maculopathy to resolve in our study is significantly shorter compared to their outcomes. They reported a range of 6–16 months (with a mean duration of 12 months) for complete resolution of the maculopathy while we achieved similar results in only 18.2 weeks (approximately 4 and half months). The most important issue to discuss with ODP-M is that although most surgical techniques have been reported to achieve desired anatomical and functional outcomes, these started to become evident after at least 3 months and complete resolution required 6–12 months from surgery^8,9,11,13,14,17,21,22,25,27,31,32^.

We believe the use of a scleral plug might aid in quicker resolution. Our results also support the findings suggested by Babu et al.^12^—which compared plug vs non-plugged techniques. They have mentioned that although the duration of SRF resolution was comparable in all groups, the central foveolar thickness returned to normal only in the eyes which were plugged—via either scleral or inverted ILM.

Results from various combination and their conclusions have been illustrated in Table 1.

**Table 1:** Results and conclusions from various studies using different combinations of surgical steps for treatment of optic disc pit maculopathy (ODP-M).

	Steps	Success rate	Conclusion
Avci et al.^13^	pars plana vitrectomy (PPV), endolaser to the temporal edge of the optic disc and C3F8 tamponade without internal limiting membrane (ILM) peeling	61.5% in 3 months 84.6% in 12 months 92.3% in 14 months	ILMP may not be required
Hirakata et al.^21^	Pars plana vitrectomy, induction of posterior vitreous detachment (PVD), and gas tamponade were performed	10 of 11 eyes had complete attachment but required nearly 1 year	Endo lasers may not be required
Hirakata et al.^11^	Pars plana vitrectomy with induction of a posterior vitreous detachment (PVD) was performed in all eyes. No laser or gas injection was performed in any eye during the original surgery	Complete retinal reattachment was achieved in 7 of 8 eyes, up to about 1 year was necessary for the retinal detachment to resolve fully	Endo laser and internal tamponade may not be required
Theodossiadis^34^	Scleral sponge of 7.5 × 5.5 mm was fixed at the posterior pole of the globe corresponding to the macula along the vertical axis of the 12-to-6 o'clock meridian. No additional treatment of any kind (laser, diathermy, or cryotherapy) was used	The absorption of the macular fluid started immediately after the operation and was completed after 5 to 6 months	Vitrectomy may not be required
Akiyama et al.^35^	Intravitreal gas injection was (SF6) performed on 8 consecutive patients	Complete retinal reattachment after only gas tamponade was achieved in four out of eight eyes The period required for reattachment after final gas treatment was 12 months	Gas alone is not beneficial
Rayat et al.^28^	PPV + PVD induction with variable adjunctive	Internal limiting membrane peel and temporal endolaser were not associated with postoperative reattachment, nor was there a difference between air and SF6 and C3F8 gas tamponade	Adjunct techniques such as internal limiting membrane peel and temporal endolaser may not improve outcomes; nor does there seem to be a difference between short- and long-acting gases Duration of recovery may take over a year
Our study	PPV with PVD induction and scleral plug (no ILMP, No Endolaser, only atmospheric air tamponade)	Earlier resolution with good outcomes in all non-complicated cases	ILM peeling, Endolaser, special tamponade may not be required Use of Scleral plug may cause earlier resolution

### Pattern of resolution

We also observed a specific pattern of resolution in all of our cases (Table 2). The resolution of the subretinal fluid was followed by the resolution of the schitic like lesions. Residual macular edema was the last to resolve in the sequence. The stages and mean duration for each of the stages are illustrated below.

**Table 2:** Various stages of resolution of optic disc pit maculopathy (ODP-M).

Stage	Findings
Stage of subretinal fluid (SRF) resolution	From the beginning of the stage where the subretinal fluid starts resolving to complete resolution
Stage of resolution of retinoschitic lesions (RL)	Subretinal fluid must not be present. From the beginning of the stage where the retinoschitic lesions start resolving to complete resolution
Stage of resolution of macular edema (ME)	Retinoschitic lesions must not be present. Resolution of the central macular thickness to normal thickness for the given age, gender and ethnicity

Various studies have already demonstrated that resolution of the subretinal fluid was the earliest finding following surgery^9,13,28^. The terminology SRF is appropriately used and is straightforward in the majority of the literature. They also do comment on how long it might require for the subretinal fluid to resolve and its correlation with functional gain.

However, when referring to RL there is found to be a deviation in use of terminologies to describe this finding. Literatures have used outer/intraretinal fluids (ORF/IRF), multilayer fluid (MLF)^12^, outer or inner layer schisis-like separation, and edema-like spaces^11,21,33^ to describe similar lesions causing an “inter- literature” confusion. We condemn the use of the term retinoschisis because the term is used to denote a near absolute disconnection between the outer plexiform and inner nuclear layers causing an absolute scotoma. Most literatures describe good visual recovery after resolution of these lesions hence making the entity contradictory. The lesions are also termed as intraretinal fluids or multilayered intraretinal fluids which is rather difficult to apprehend due to the variation of their use in different contexts. We hence propose a term “pseudoschisis” or “retinoschitic lesions” to refer to such lesions to maintain uniformity for future references.

ME was found to be the last to resolve in the sequence of recovery. This was found to be in congruency with many reports from the literature which have noted the central macular thickness stabilized only in the later stages. A stable and appropriate central macular thickness via OCT analysis for the given age, gender and ethnicity has often been used an indicator for a successful recovery and may still hold the ground as a predictable indicator. Although it is beyond the scope of this paper to comment on the functional gain, one must continue considering various other factors—such as the status of the retinal pigment epithelium, ellipsoid layer, and presence of outer retinal tubulations, etc. to prognosticate functional outcome.

## Conclusion

Surgical intervention limited to only PPV with PVD induction and scleral tissue plug for treatment of ODP-M without adjunctive procedures like ILM peeling, laser or using long term tamponading agents can produce comparably good results in cases of ODP-M. The use of scleral plugs may hasten resolution. ILM peeling, endolaser, long-term tamponade with expansile gases or head positioning may not require to be performed routinely and can be reserved for complicated cases.

Understanding the pattern of resolution along with the duration that is required for the lesions to start demonstrating signs of recovery may vary. The pattern of resolution was found to be in the order of disappearance of SRF, followed by resolution of the RL and then ME. Use of specific terminologies to define various lesions can enhance uniformity when describing/referring to an ODP-M.

### Limitation of the study

This study is an exploratory study and further multicenter studies must be performed to support the results. Also, the pattern of resolution only reflects results of only those eyes which did not have complicated ODP-M—we did not include eyes with lamellar or full-thickness macular holes or abnormalities associated with ILM surfaces like epiretinal membranes or macular puckering. A study to understand the pattern of resolution in other surgical techniques, outcomes of the procedure if vitreous cavity is left filled with balanced salt solution and also the inclusion of complicated ODP-M to understand can further enhance the scope of these novel findings.

### Supplementary Information

Supplementary Legends.

Supplementary Video 1.

## Data Availability

The data associated with the study will be made available from the corresponding author on reasonable request.
