# Supplementary material for: Minimally invasive procedure for optic disc pit maculopathy: vitrectomy with scleral plug and analysis on pattern of resolution
Source: Sci Rep. 2023 Sep 21;13:15724. doi: 10.1038/s41598-023-42839-y (PMC10514184; doi:10.1038/s41598-023-42839-y)
Supplement: Supplementary file 1 — Supplementary Legends. [file 41598_2023_42839_MOESM1_ESM.docx]

Supplementary Video 1: The video illustration of the minimally invasive procedure for management of optic pit maculopathy. Note the scleral graft was harvested and inserted into the optic pit. The highlight of the procedure is on how a successful outcome can still be achieved without any additional procedures such as internal liming membrane peeling , parapapillary photocoagulations or use of long term tamponading agents.
